# Visual Performance of Two Designs of Myopia Management Soft Contact Lenses Compared with a Monofocal One in Young Adults

**DOI:** 10.18502/jovr.v18i4.14544

**Published:** 2023-11-30

**Authors:** Isabel Signes-Soler, Silvia Roselló Sivera, Javier Cantó-Vañó, Inmaculada Giménez-Sanchís, César Albarrán-Diego

**Affiliations:** ^1^Universidat de València, Department of Optics and Optometry and Vision Sciences, Burjassot, Valencia, Spain; ^2^Optica Signes, Calpe (Alicante); ^3^Optica Claravisión Ontinyent, Valencia, Spain; ^4^Clínica Dr Gonzalo Muñoz, Valencia, Spain; ^6^https://orcid.org/0000-0001-5525-9392

**Keywords:** Dual-focus contact lens, Extended depth of focus contact lens, Myopia control

## Abstract

**Purpose:**

To compare the visual performance of two distinct types of soft contact lenses (CL) aimed at slowing down myopia progression with the performance of a monofocal soft CL.

**Methods:**

In a prospective double-masked, crossover trial, 18 myopic adults (aged 18–30 years old) were fitted in a randomized order with three types of disposable CL: MiSight ${}^{\text{TM}}$ (dual-focus), Mylo ${}^{\text{TM}}$ (extended depth of focus -EDOF-), and Clariti ${}^{\text{TM}}$ (single distance vision). Measurements were taken after wearing the CL for five days with five days off in between at two different optometry centers. High contrast distance visual acuity (VA) with spectacles and for each of the different CL, subjective refraction, slit lamp exam, aberrometry, stereopsis, monocular and binocular amplitude of accommodation and accommodative facility, and horizontal phorias were measured.

**Results:**

The high contrast distance VAwas better for the single vision CL compared to the myopia control CL. No significant differences were observed between the r two myopia control CL. The overall root mean square (RMS) was higher for the double focus CL (RMS = 1.18 ± 0.29 µm), followed by the EDOF CL (RMS = 0.76 ± 0.35 µm) and then the single vision CL (RMS = 0.50 ± 0.19 µm). The primary spherical aberration (SA) mean value was low for all of the three CL, without statistical differences among them. No other significant differences were detected.

**Conclusion:**

The overall RMS resulted in a higher value for the dual-focus than the EDOF CL, but no differences in high contrast distance VA and binocularity were detected between them. The monofocal CL's performance was better than the myopia control CL.

##  INTRODUCTION

Myopia is becoming a growing concern for public health due to its increase in prevalence worldwide and its association with several ocular problems such as retinal detachment, choroidal degeneration, cataracts, and glaucoma.^[1]^ It is estimated that by 2050, 50% of the world population will be myopic with 9.8% of these future myopes, approximately 938 million people, being highly myopic.^[2,3]^ Risk factors for high myopia (spherical equivalent ≤ —6.00 D or an axial length ≥ 26.5 mm) in adulthood include parental myopia, age at baseline, myopic progression during the first year after onset, increased time spent on reading and close-up work, and less outdoor activity during childhood.^[4]^ The major concern with high myopia is pathologic myopia, which is excessive axial elongation associated with myopia that leads to structural changes in the posterior segment of the eye, such as posterior staphyloma, myopic maculopathy, and high myopia-associated optic neuropathy. This can result in loss of best-corrected visual acuity (VA).^[5]^ While posterior staphyloma has been found in eyes with normal axial length (24 mm), it could indicate that factors other than axial length elongation may influence the occurrence of pathologic myopia.^[6]^ However, axial elongation remains an important risk factor in pathologic myopia, and several interventions have been proposed and tested in recent years to slow down the axial elongation of the eye.

These interventions included pharmacological treatments based on administration of low-dose atropine, and/or optical treatments based on the hypothesis of approaching peripheral blur, where special designs of ophthalmic and contact lenses, or orthokeratology were used.^[7,8,9]^ Among these treatments, when compared to orthokeratology lenses, soft myopia control lenses have risen in popularity for their ease of fitting and their ease of handling in children. Soft myopia control lenses are based on producing a certain myopic peripheral defocus^[10]^ that is hypothesized to be the reason for the slowing down of myopia.

Since myopia control soft CL-induced peripheral image blur, it would be logical to think that they may also contribute toward some degree of decrease in visual performance.^[11]^ This study aims to compare the visual performance of two types of CL used for myopia control, that is, a dual-focus CL (Misight ${}^{\text{TM}}$ , CooperVision) and an EDOF soft CL (Mylo, ${}^{\text{TM}}$ Mark'ennovy) with the performance of a single-vision CL (Clariti 1 day ${}^{\text{TM}}$ , CooperVision). To the best of our knowledge, this is the first independent study that compares the performance of these two types of CL with that of a single vision CL.

**Figure 1:**
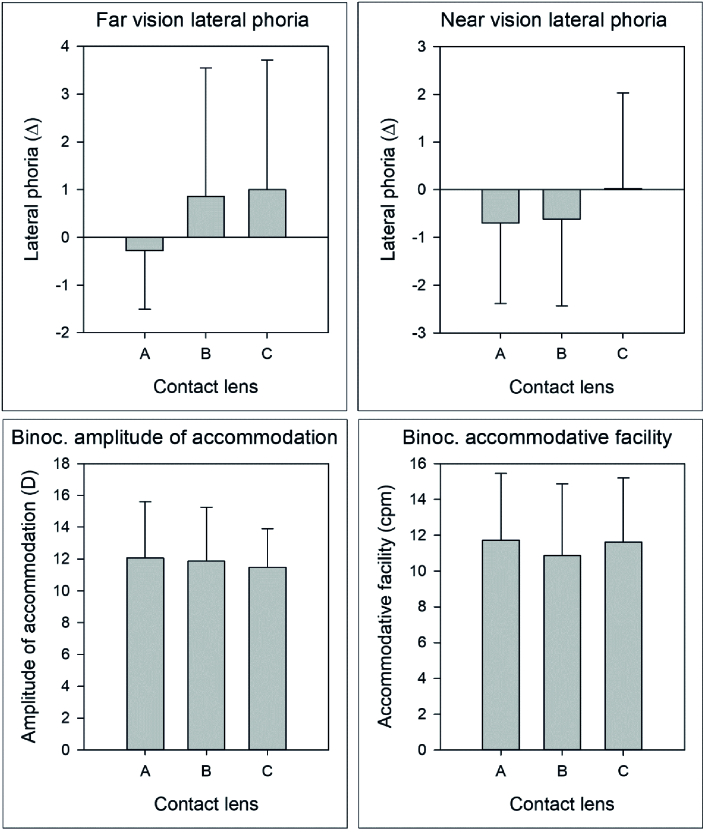
Monocular accommodative amplitude (bottom left) and accommodative facility (bottom right). Far and near vision lateral phoria (top right and left).

**Figure 2:**
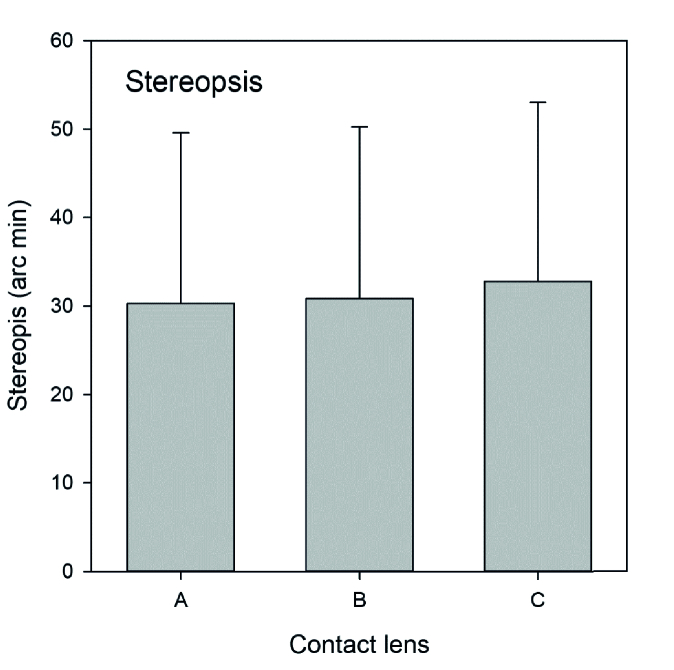
Steoreopsis for the 3 CL.

**Table 1:** Visual acuity (VA) data for the three types of contact lenses.


	orange**Mean**	orange**STD**	orange**Min**	orange**Max**
**Age (years)**	23.83	3.73	18	30
**Sex**	<@13 women		<@5 men	
**Occupation**	<@8 workers		<@10 students	
**Sph (D)**	- 2.63	1.25	- 1.00	- 4.50
**Cyl (D)**	- 0.26	0.29	0.00	- 0.75
**SE (D)**	- 2.76	1.31	- 1.00	- 4.50
	
	
white<bcol>5</ecol>Sph, sphere; Cyl, cylinder; SE, spherical equivalent, D, diopter, STD, standard deviation; Min, minimum; Max, maximum

**Table 2:** Wavefront mean quadratic errors (RMS) and spherical aberration without contact lens (NC) and with contact lens A, B, and C, for a 5 mm pupil.


	orange**RMS (µm)**				orange**Spherical aberration (µm)**			
	orange**Mean**	orange**STD**	orange**Min**	orange**Max**	orange**Mean**	orange**STD**	orange**Min**	orange**Max**
**NC**	2.87	1.04	0.73	4.33	0.038	0.076	- 0.096	0.177
**CL-A**	0.50	0.19	0.23	0.85	- 0.040	0.082	- 0.229	0.102
**CL-B**	1.18	0.29	0.77	1.76	0.033	0.106	- 0.131	0.231
**CL-C**	0.76	0.35	0.35	1.47	- 0.035	0.082	- 0.218	0.068
	
	
white<bcol>9</ecol>NC, no contact lens; CL-A, Contact lens A (Clarity 1day); CL-B, Contact lens B (Misight); CL-C, Contact lens C (Mylo)

**Table 3:** Vertical and horizontal coma aberration data for a 5 mm pupil.


	orange**Coma (mm)**			
	orange**Mean**	orange**STD**	orange**Min**	orange**Max**
**NC**	V	0.029	0.119	- 0.147	0.305
	H	0.019	0.080	- 0.130	0.171
**CL-A**	V	0.002	0.105	- 0.270	0.153
	H	-0.003	0.095	- 0.224	0.160
**CL-B**	V	0.039	0.096	- 0.113	0.183
	H	0.018	0.102	- 0.136	0.209
**CL-C**	V	0.025	0.110	- 0.138	0.215
	H	-0.043	0.076	- 0.159	0.159
	
	
white<bcol>6</ecol>NC, no contact lens; CL-A, Contact lens A (Clarity 1day); CL-B, Contact lens B (Misight); CL-C, Contact lens C (Mylo)

##  METHODS

This is a prospective, double-masked, randomized multicenter crossover study. Participants signed written informed consent and the study was conducted in accordance with the tenets of the Helsinki Declaration and was approved by the Ethics Committee of Research in Humans of the Ethics Commission in Experimental Research of the University of Valencia (Spain) with register code 1575195.

### Subjects

Young myopes from two private optometric practices in Spain, one in Calpe (Alicante, Spain) and one in Ontinyent (Valencia, Spain) were invited to participate in the study. Data were collected between January and September 2021. A total of 26 participants met the inclusion criteria on the dates set for the study and were enrolled in the study. Of these, 18 (13 females and 5 males) with mean age of 23.83 ± 3.72 years (aged 18–30 years old) completed the study, nine from each practice. All individuals were invited to participate in the study regardless of their gender. It is possible that women were more willing to collaborate or had easier access due to the study location or their job. The inclusion criteria were as follows: participants had to be between the ages of 18 and 30, with a distance corrected VA of 20/25 or better, a refractive error between –0.50 and –6.00 D, and astigmatism of ≤ 0.75 D. Additionally, they should not have a history of any ocular or binocular vision disorder or any systemic condition. Previous experience with CL usage was not necessary. Selected participants underwent a slit lamp examination to ensure there was no ocular pathology, and a direct ophthalmoscopy confirmed the absence of retinal pathology. All participants were required to sign an informed consent and attend follow-up visits.

### Masking Procedure

There were two optometrists involved in each center, one unmasked and the other masked. The unmasked optometrist was responsible for executing the first review of the participants, to assess whether they met the requirements for inclusion into the study. Objective and subjective refraction were performed. Once they met the inclusion criteria and their participation in the study had been accepted, this optometrist randomly (https://www.randomizer.org) chose and fitted a pair of CL. After slit lamp examination and VA verification, the lenses were used by the participant for 5 days. The CL were marked with a D (for right) or I (for left) to mask participants to the lens type. The masked optometrist then performed the optometric examination after 5 days without knowing what CL the participant was using. The unmasked optometrist then gave the participant another randomly chosen CL and the same procedure was followed for each CL.

### CL and Fitting

Three different types of CL were fitted for all the participants in the study, one of them was a monofocal standard CL (Clariti, CooperVision), while the others were a dual-focus myopia control CL (Misight, ${}^{\text{TM}}$ CooperVision) and an EDOF control myopia CL (Mylo, ${}^{\text{TM}}$ Mark'ennovy). The washout period was at least one week, but in some cases, it was several weeks depending on the participant's availability.

The Clariti 1-day ${}^{\text{TM}}$ (CooperVision) is a daily disposable silicone hydrogel CL made of somofilcon A material, with an 8.6 mm base curve, 14.1 mm diameter, center thickness (at –3.00DS) of 0.07 mm, and Dk/t (at –3.00DS) of 86.

The MiSight ${}^{\text{TM}}$ (CooperVision) is a daily disposable soft CL which has already been described in other articles.^[11,13]^ It has been reported to slow down myopia progression in spherical equivalent refraction by 39.3% over 2 years^[14]^ and 59% over 3 years,^[13]^ with minimal impact on ocular physiology in children,^[15]^ minimising the myopic axial elongation but retaining the underlying physiological elongation observed in emmetropic eyes.^[16]^

The Mylo ${}^{\text{TM}}$ (Mark'ennovy) is a silicone hydrogel CL (Filcon 5B) with an extended depth of focusdesign, powered by the Brien Holden Vision Institute's patented EDOF technology^[17]^ and has been described by Rizzo et al.^[18]^ It has been proven to reduce myopia up to 43% after 2 years of usage,^[19]^ with no rebound effect after this time.^[20]^

The CL fit was assessed by the unmasked optometrist to ensure an acceptable fit before dispensing the CL. The manufacturer's recommendations were followed for the fitting process. Once the CL was chosen, its centration, adequate movement, over-refraction ( < 0.25D) and satisfactory VA ( ≤ 0.00 logMAR in each eye) were all evaluated by the masked optometrist.

### Measurement Procedure 

Measurements were taken before starting the study without CL insertion and then after using each of the three CL. Each participant attended four scheduled visits (including the baseline visit). Measurements comprised subjective refraction, slit lamp exam, i-profiler aberrometry, stereopsis, accommodative facility and amplitude, and phorias. The distance CL power was based on the corrected distance refraction (spherical equivalent), and participants were over-refracted each time to adjust for any errors in spherical refraction.

High contrast distance VA was measured at 5m using a backlit chart, and at 40 cm with the ETDRS logarithmic VA chart. The ocular wavefront aberrometer i.Profiler *${}^{\text{plus}}$* (Carl Zeiss Vision, Germany) was used to measure the aberrations. Measurements were taken prior to the adaptation of the CL and then with CL A (Clariti 1-day ${}^{\text{TM}}$ CooperVision), B (Misight ${}^{\text{TM}}$ CooperVison), and C (Mylo ${}^{\text{TM}}$ Mark'ennovy), after 5 days of use. We recorded the data of the total aberrations by means of the root mean square (RMS) value, and also the primary spherical aberration Zernike coefficient ${(Z}_{4}^{0}$ ).

Lateral phoria was measured using cover test and compensated with Risley prism (horizontal) at 5 m and 40 cm, until it showed orthophoria. The push-up method was used to measure monocular and binocular amplitude of accommodation, approaching the EDTR test to the participant's eye until blurring occurred, and converting this distance (in m) into vergence (in D). Binocular and monocular accommodative facility was tested with +2.00/-2.00 D flippers to assess the dynamics of the accommodative system. The test was administered at 40 cm and measured in cycles per minute (cpm), using an EDTR test. For stereopsis the Randot stereotest was used with 10 levels of disparity (400 to 20 seconds of arc at 40 cm).

### Statistical Analysis

Data analysis were performed using the SigmaPlot for Windows version 14 software (Systat Software, Inc., UK). Descriptive data were provided as means ± standard deviations (STD), and maximum and minimum values. The normality of the data was checked using the Shapiro Wilk test. The one-way repeated measures analysis of variance (ANOVA) was performed to detect differences among groups (or the corresponding Friedman repeated measures analysis of variance on ranks test, when the data failed the normality test). Post hoc testing to locate differences, when found with the ANOVA test, was conducted by means of the Holm-Sidak method. P-values < 0.05 were considered statistically significant.

##  RESULTS

### Sample

Data from 18 subjects were obtained. Of these, 72% of the sample were female (Fisher exact test, *P* = 0.018). Only right eye values were considered for statistical analysis of monocular data. The results obtained for the percentage of students (56%) and workers (44%) were similar (Fisher exact test, *P* = 0.740). The mean ± SD sphere, cylinder, and spherical equivalent were - 2.63 ± 1.25 (range - 1.00 to - 4.50), - 0.26 ± 0.29 (range 0.00 to - 0.75) and - 2.7 ± 1.31 (range - 1.00 to - 4.50), respectively.

### High Contrast Visual Acuity

The ANOVA test revealed statistically significant differences in VA among the four compensation options: spectacles, CL A, CL B, CL C (*P* = 0.004). Post hoc testing by the Holm-Sidak method located these differences in the pairwise comparisons CL-A vs CL-B (*P* = 0.012) and CL-A vs CL-C (*P* = 0.032), with no differences for the rest of comparisons in high contrast VA. Monocular VA achieved with spectacles was similar to that achieved with any of the CL. Among the three CL considered, CL-A provided better VA, similar to the average VA provided by spectacles, but with a maximum VA higher in the group of CL-A. Table 1 shows VA for spectacles, and the three types of CL.

### Total Aberrations

Table 2 shows the wavefront mean quadratic errors, considering all aberrations (lower and higher order). Data was provided for eyes without compensation (NC), that was with the naked-eye and for compensation with each of the three CL considered.

The overall RMS, considering lower and higher order aberrations together, was obviously higher for the no-compensation option. Excluding this situation from the analysis and considering only the three CL compensation options, the ANOVA test revealed statistically significant differences in RMS among the three CL (*P* < 0.001). Post hoc testing by the Holm-Sidak method located those differences in all the possible pairwise comparisons CL-A vs CL-B (*P* < 0.001), CL-A vs CL-C (*P* = 0.003), and CL-B vs CL-C (*P* < 0.001). Therefore, it can be said that the aberrometric profiles of the eyes compensated with the three CL were different, with a lower value for the CL-A (RMS = 0.50 ± 0.19 µm), followed by the CL-C (RMS = 0.76 ± 0.35 µm), and with the highest values for the CL-B (RMS = 1.18 ± 0.29 µm).

### Spherical Aberration

Spherical aberration data did not pass the normality testing (Shapiro-Wilk Test, *p* < 0.050), so the non-parametric Friedman repeated measures analysis of variance on ranks was applied revealing statistically significant differences (*P* < 0.001). The Tukey post hoc testing procedure located these differences in the comparisons NC vs CL-A (*P* = 0.001), and NC vs CL-C (*P* = 0.003), with no differences for the rest of the comparisons, that is between NC and CL-B and between the 3 types of CL. The spherical aberration mean value was low for the three CL, being slightly positive for the CL-B, and slightly negative for both the CL-A and CL-C. If the spherical aberration comparison was repeated excluding the no-compensation data, no significant differences were observed among the three CL (*p* = 0.054). Table 2 shows primary spherical aberration data isolated from the rest of aberrations, by means of the corresponding Zernike coefficient ${(Z}_{4}^{0}$ ).

### Coma Aberration

The two-way repeated measures analysis of variance was applied to the data, with orientation of coma being one of the factors (with two levels: vertical and horizontal) and compensation being the other factor (with 4 levels: no compensation, compensation with CL-A, with CL-B, and with CL-C). No significance was observed for any of the two factors (*P* = 0.381 for coma orientation, and *P* = 0.088 for compensation), and no interactions between factors were found (*P* = 0.089). Table 3 shows coma aberration data isolated from the rest of the aberrations, by means of the corresponding Zernike coefficients (Z3-1 for vertical coma, and Z3+1 for horizontal coma) for a 5 mm pupil.

### Monocular Accommodation Values

Figure 1 shows mean ± STD values for monocular accommodative amplitude (bottom left) and accommodative facility (bottom right). No statistically significant differences were observed among the three CL, either for the monocular amplitude of accommodation (ANOVA, *P* = 0.551), or for the monocular accommodation facility (ANOVA, *P* = 0.093).

### Binocularity

No differences were observed among the three tested CL in far vision phoria (Friedman repeated measures analysis of variance on ranks, *P* = 0.069), near vision phoria (*P* = 0.155), binocular amplitude of accommodation (ANOVA, *P* = 0.436), binocular accommodative facility (*P* = 0.343), or stereopsis (*P* = 0.584).

Figure 1 shows far- and near-vision lateral phoria and amplitude of accommodation and accommodative facility (top right and left).

The mean stereopsis measured in seconds of arc was 30.28 ± 19.29, 30.83 ± 19.42, and 32.78 ± 20.24 for monofocal, dual-focus, and EDOF CL respectively, as shown in Figure 2.

##  DISCUSSION

This clinical trial was designed to assess the visual performance of two types of soft CL used for controlling myopia progression, MiSight (dual-focus) and Mylo ${}^{\text{TM}}$ (EDOF), which were both compared to a monofocal CL, Clariti, in a young cohort of myopic participants. The EDOF CL used in our study is a lens recently marketed by *Mark'ennovy* as Mylo ${}^{\text{TM}}$ with studies performed only at the prototype stage.^[17,19]^

Visual performance of a dual-focus CL compared to a multifocal CL in young adults has been reported in prior studies.^[11]^ There are also studies on the visual performance of some EDOF CL prototypes for high and low addition,^[12]^ but as far as we are aware there are no previous studies comparing the dual-focus CL with a customizable EDOF CL (medium addition, +1.5 D).

For the purpose of advising parents and children who are considering soft CL as a strategy for myopia control, vision care professionals need to understand how this type of CL performs. Regarding its safety, children using MiSight over a 6 year period had no serious ocular adverse events.^[15]^ Today, the effect on the delay in the progression of myopia is no longer debatable.^[13,19,21]^ However, we need to assess what can be expected when wearing these types of CL in terms of visual quality and their effects on binocular vision.

### High contrast VA.

During the assessment of high contrast VA, noticeable differences were detected between the monofocal CL and both of the myopia control CL. In contrast, the VA was found to be less satisfactory with the myopia control CL.

These outcomes coincide with research findings reported by Garcia-Marqués et al regarding high-contrast VA between dual-focus and single vision CL, measured 25 min after insertion.^[22]^ However, Sha et al found a significantly better high VA with the dual-focus CL when compared to two prototypes of EDOF CL, nevertheless these EDOF CL were subjectively better tolerated.^[12]^ Prototype 1 (low addition) and prototype 2 (high addition) had different levels of addition than that of Mylo ${}^{\text{TM}}$ (medium addition +1.5D). In fact, prototype 1 was significantly better than prototype 2 in terms of its binocular low contrast VA for distance measurement. The addition of the lens plays an important role on its performance.

In 2013, Kollbaum and colleagues discovered that the dual-focus contact lens yielded favorable VA results. The achieved VA values were comparable to those seen in lenses that achieved good VA through conventional multifocal lens correction methods. However, these authors pointed out the possible decrease in visual performance, similar to that experienced with other CL containing multiple refractive zones.^[11]^ When compared to a multifocal CL for presbyopia (Acuvue Oays) in presbyopic participants, EDOF lenses resulted in significantly better high contrast VA in intermediate and near vision but not at distance.^[23]^ Although the measurements were obtained after only 10 minutes of settling time, it is important to consider that the participants in this cohort study were presbyopic while we tested the young adults. Although the cohort is different, as these were presbyopic participants and we tested young adults, the measurements were also done after only 10 min of settling time. Extending the adaptation period for multifocal soft CL can lead to notably improved high contrast VA.^[24]^ Furthermore, it is important to note that this brief-term study might not accurately predict the complete capabilities of extended depth of focus contact lenses (EDOF CLsCL), as limited usage durations could obscure certain outcomes.

### Binocularity

No significant difference was observed in any of the CL for stereopsis at 40 cm. Similar to these outcomes, Sha et al,^[17]^ did not find any significant differences in stereopsis between participants fitted with dual-focus and those fitted with EDOF CL. They observed poorer stereopsis than those in our study (over 30 s of arc for any of the CL). In the same way, when comparing the dual-focus with the monofocal CL (Proclear), no differences were observed in terms of stereopsis for short-term use of the CL in young myopic adults^[22]^ and children.^[25]^ Nevertheless, Hiraoka et al, reported a significantly better stereopsis with EDOF CL (1 day Pure) when compared to a single vision CL, but their study was done on a very specific population, on eyes that had undergone monofocal intraocular lens implantation.^[26]^ In fact, our mean value for the dual-focus lens (30.83 ± 19.42) was very similar to that found by Garcia-Marques et al.^[22]^

Chen and colleagues correlated reduced accommodative response with greater myopia progression within the group with positive spherical aberration, suggesting that some subjects used the positive spherical aberration as an accommodative response for near vision, inducing hyperopic defocus at the retina.^[27]^ In our study, we did not find any significant difference in accommodating among the various CL, despite their distinct optical designs. Dual-focus achieved a significantly lower monocular accommodative facility when compared to the two EDOF prototypes in the work by Sha et al,^[17]^ while in our study the differences were not significant.

Throughout our study, no significant differences were detected in accommodation among the various contact lenses, despite their distinct optical designs.

A recent paper has reported the dynamics of the accommodative response and facility with MiSight when compared to a single vision soft CL (Proclear) in a similar cohort of young adults. The authors found higher variability and greater lags of accommodation with the dual-focus at near distances. In addition, a worse quantitative facility performance was observed with the MiSight when compared to the single vision lenses.^[28]^ Within our study, we identified no notable distinctions among the three types of LCs regarding either accommodation or facility. Our assessment was subjective, in contrast to the prior study which employed an open-field autorefractometer for measurement. On the other hand, Gifford et al, compared the same CL detected accommodative responses after 10 min adaptation with no lag using the same autorefractor. They also assessed the refraction instability and the dual-focus showed greater instability than Proclear.^[29]^

Children aged between 10 and 15 years wearing multifocal CL showed reduced accommodative responses and more exophoria at 40 cm with increasingly higher accommodative demands than with single vision Clin a study conducted by Gong et al.^[30]^ This suggests that children may be relaxing their accommodation and using the positive addition or increased depth of focus from added spherical aberration of the multifocal CL. Nevertheless, they did not find any significant differences in accommodative amplitude or facility, which coincides with our results. Further studies are needed to evaluate other lens designs, application of variable amounts of positive addition, the existence of aberrations, and long-term adaptation to lenses.

Exophoria was regarded as having negative values, while endophoria was treated as having positive values, allowing us to quantify them accordingly. As shown in Figure 1, with the monofocal CL, the distance vision lateral phoria was slightly exophoric and the two-myopia control CL was endophoric with no significant differences among the three. Cheng et al^[27]^ found significant differences at first week for distance phoria but they compared a single vision CL to a positive spherical aberration CL.

### Aberrations

The total RMS of the dual-focus CL (RMS = 1.18 ± 0.29 µm) were significantly higher than those of the EDOF CL (RMS = 0.76 ± 0.35 µm). This suggests that the dual-focus induces greater aberrations. Higher order aberrations were also higher for the dual-focus as compared to a single vision CL (Proclear) in a young adult cohort.^[22]^ Some EDOF prototypes have demonstrated reduced aberrations when compared to multifocal designs like concentric bifocal dual-focus,^[31]^ which is in accordance with our results. The observed increase in total aberrations induced by the use of the dual-focus contact lens might be associated with improved subjective comfort and quality of vision reported with the extended depth of focus contact lens (EDOF CL), as indicated by certain studies.^[12,32]^

In our study, no significant differences were observed in the amount of spherical aberration measured among the three CL tested, with the dual-focus CL inducing a small value of positive spherical aberration, whereas the standard monofocal and the EDOF CL produced small values of negative spherical aberrations. An increase in the spherical aberration with center-distance multifocal soft CL was found by Fedtke et al.^[33]^ For a 5 mm pupil, the dual-focus CL design produced an increase in positive spherical aberration depending on the addition.^[11,34,35]^ These findings should also be assessed together with comprehensive questionnaires to investigate whether participants subjectively perceive these differences.

We are aware of the limitations of our study. Larger sample size studies could lead to clearer conclusions. The contrast sensitivity measure would have provided more accurate information on visual quality than the conventional high contrast VA measurement utilized in our study. Certainly, contrast sensitivity was documented, but its calculation varied across the different centers due to the use of distinct methods. As a result, the outcomes were omitted from the analysis. The two optometric centers that have participated in the study are independent and have no relationship with the CL industry, which guarantees impartiality when presenting the results.

In summary, the present study compares the performance of two types of myopia control CL, a dual-focus CL and an EDOF CL with the performance of a single vision CL. The single vision CL showed slightly better VA results as compared with each of the two myopia control CL, with no differences between the latter two. No differences were found either among the three CL in far and near vision phoria, binocular amplitude of accommodation, binocular accommodative facility or stereopsis.

However, the aberrometric profile of the eyes compensated with the three CL was different. The overall RMS, considering lower and higher order aberrations together, was higher for the dual-focus, followed by the EDOF and then the single vision CL. This could explain improved comfort and visual performance with the EDOF which should be verified in future studies. No significant differences in spherical aberration were found among the CL tested. A minor decrease in high contrast distance VA might be anticipated when using CL designed to slow down myopia progression. However, this reduction is expected without causing any disruption to the vergence or accommodative systems.

##  Financial Support and Sponsorship

None.

##  Conflicts of Interest

None.
